# A comparative study based on image quality and clinical task performance for CT reconstruction algorithms in radiotherapy

**DOI:** 10.1120/jacmp.v17i4.5763

**Published:** 2016-07-08

**Authors:** Hua Li, Steven Dolly, Hsin‐Chen Chen, Mark A. Anastasio, Daniel A. Low, Harold H. Li, Jeff M. Michalski, Wade L. Thorstad, Hiram Gay, Sasa Mutic

**Affiliations:** ^1^ Department of Radiation Oncology Washington University St. Louis MO; ^2^ Department of Biomedical Engineering Washington University St. Louis MO; ^3^ Department of Radiation Oncology University of California Los Angeles CA USA

**Keywords:** ALARA principle, task‐based image quality, ${\text{iDose}}^{4}$ reconstruction, simulation CT, radiotherapy

## Abstract

CT image reconstruction is typically evaluated based on the ability to reduce the radiation dose to as‐low‐as‐reasonably‐achievable (ALARA) while maintaining acceptable image quality. However, the determination of common image quality metrics, such as noise, contrast, and contrast‐to‐noise ratio, is often insufficient for describing clinical radiotherapy task performance. In this study we designed and implemented a new comparative analysis method associating image quality, radiation dose, and patient size with radiotherapy task performance, with the purpose of guiding the clinical radiotherapy usage of CT reconstruction algorithms. The ${\text{iDose}}^{4}$iterative reconstruction algorithm was selected as the target for comparison, wherein filtered back‐projection (FBP) reconstruction was regarded as the baseline. Both phantom and patient images were analyzed. A layer‐adjustable anthropomorphic pelvis phantom capable of mimicking 38–58 cm lateral diameter‐sized patients was imaged and reconstructed by the FBP and ${\text{iDose}}^{4}$ algorithms with varying noise‐reduction‐levels, respectively. The resulting image sets were quantitatively assessed by two image quality indices, noise and contrast‐to‐noise ratio, and two clinical task‐based indices, target CT Hounsfield number (for electron density determination) and structure contouring accuracy (for dose‐volume calculations). Additionally, CT images of 34 patients reconstructed with ${\text{iDose}}^{4}$ with six noise reduction levels were qualitatively evaluated by two radiation oncologists using a five‐point scoring mechanism. For the phantom experiments, ${\text{iDose}}^{4}$ achieved noise reduction up to 66.1% and CNR improvement up to 53.2%, compared to FBP without considering the changes of spatial resolution among images and the clinical acceptance of reconstructed images. Such improvements consistently appeared across different ${\text{iDose}}^{4}$ noise reduction levels, exhibiting limited interlevel noise (<5 HU) and target CT number variations (<1 HU). The radiation dose required to achieve similar contouring accuracy decreased when using ${\text{iDose}}^{4}$ in place of FBP, up to 32%. Contouring accuracy improvement for ${\text{iDose}}^{4}$ images, when compared to FBP, was greater in larger patients than smaller‐sized patients. Overall, the ${\text{iDose}}^{4}$ algorithm provided superior radiation dose control while maintaining or improving task performance, when compared to FBP. The reader study on image quality improvement of patient cases shows that physicians preferred ${\text{iDose}}^{4}$‐reconstructed images on all cases compared to those from FBP algorithm with overall quality score: 1.21 vs. 3.15, p=0.0022. However, qualitative evaluation strongly indicated that the radiation oncologists chose ${\text{iDose}}^{4}$ noise reduction levels of 3–4 with additional consideration of task performance, instead of image quality metrics alone. Although higher ${\text{iDose}}^{4}$ noise reduction levels improved the CNR through the further reduction of noise, there was pixelization of anatomical/tumor structures. Very‐low‐dose scans yielded severe photon starvation artifacts, which decreased target visualization on both FBP and ${\text{iDose}}^{4}$ reconstructions, especially for the 58 cm phantom size. The ${\text{iDose}}^{4}$ algorithm with a moderate noise reduction level is hence suggested for CT simulation and treatment planning. Quantitative task‐based image quality metrics should be further investigated to accommodate additional clinical applications.

PACS number(s): 87.57.C‐, 87,57.Q‐

## I. INTRODUCTION

The filtered back‐projection (FBP) algorithm has been widely utilized as the standard image reconstruction algorithm for commercial CT scanners.^(1)^ Although it is computationally efficient, the FBP algorithm does not account for the stochastic nature of projection data and can produce streaking artifacts, especially for projection data with severe noise contamination. Moreover, such conditions are likely to produce inaccurate estimates of CT Hounsfield numbers. Recently, iterative reconstruction techniques have been developed to more effectively mitigate image artifacts and reduce image noise.^2^, ^3^ In radiotherapy, CT simulation images have been frequently utilized in specific tasks, including determination of tissue electron density (via CT Hounsfield numbers) and delineation of tumor and normal organ anatomy (by either manual techniques or increasingly by the use of semiautomated or automated techniques).^4^, ^5^, ^6^, ^7^, ^8^ However, the relationship between the image quality improvements of iterative reconstruction and the performance of specific radiotherapy tasks has not been fully investigated as yet.

The ${\text{iDose}}^{4}$ iterative reconstruction algorithm (Philips Medical, Cleveland, OH) has been commercially released as an artifact and radiation dose reduction tool, and mainly is used to improve image quality combined with dose reduction capabilities for diagnostic imaging purpose, while following the as‐low‐as‐reasonably‐achievable (ALARA) principle.^3^, ^9^, ^10^, ^11^ It has been reported from routine works of our clinical institutions that traditional diagnostic image quality definitions may not be realistic enough to determine the performance of the radiotherapy tasks, which certainly influence treatment planning accuracy. Inspired by a recent paradigm shift of image quality assessment that image quality should be defined by the ability of a user to perform medically or scientifically relevant tasks with the image data,^12^, ^13^ the purpose of this study is: 1) to conduct new comparative experiments of the FBP and ${\text{iDose}}^{4}$ algorithms, with varying noise reduction levels, by associating the ALARA principles with radiotherapy task performance, and 2) to provide suggestions for clinical radiotherapy usage of the ${\text{iDose}}^{4}$algorithm. This study is expected to fill the gap between image quality assessment and the clinical usage of CT simulation images in radiotherapy.

## II. MATERIALS AND METHODS

### A. Description of the phantom

**Figure 1:**
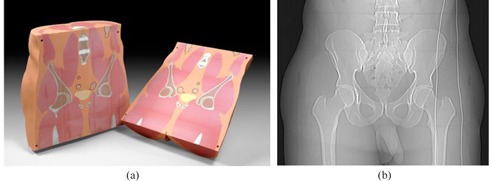
Anthropomorphic pelvis phantom (a) (courtesy to CIRS Inc., Norfolk, VA); (b) CT anterior‐posterior surview image of the phantom.

An anthropomorphic human male pelvis phantom (CIRS Inc., Norfolk, VA), as shown in Fig. 1, with additional layers of attenuation, was employed to simulate adult male patient lateral diameters of 38, 43, 48, 53, and 58 cm. The pelvis phantom was constructed from proprietary epoxy materials that mimic human tissue density and radiation attenuation properties of human tissue within 1% from 50 keV to 25 MeV. The overall phantom dimensions were 38 cm wide, 26 cm thick, and 37 cm tall. The phantom contained anatomically precise bone, cartilage, spinal cord, vertebral disks, muscle, intestines, bladder, prostate, rectum, and interstitial fat. All anatomical dimensions of this phantom were based on the visible human project datasets that serve as a reference for the study of human anatomy and are available through the National Library of Medicine. The additional attenuation layers were provided by tissue‐equivalent bolus material (Mick Radio‐Nuclear Instruments, Inc. Mount Vernon, NY), which was a synthetic oil gel with a specific gravity of 1.02, and based on vinyl plastic containing a large amount of diisodecyl phthalate.

### B. CT Acquisitions of the phantom

CT scans were acquired with a Philips Brilliance 64 slice CT simulator (Philips Medical, Cleveland, OH). To assure that the scans did not exceed the tube current limits of the scanner, we employed the commercial 4D CT protocol, which reduced the pitch to 0.06 and provided effective mAs up to three to four times the standard helical protocol. The untagged 4D CT scans were reconstructed to simulate higher dose level helical CT scans for the analysis performed in this study. Tube potentials lower than 120 kVp were not suitable for scanning the smallest phantom size of 38 cm, and certainly not any larger sizes due to higher tissue attenuation.^(14,15)^Each sized phantom was scanned with two tube potential settings, 120 and 140 kVp. The tube current modulation function was disabled in order to evaluate the image quality under the full range of effective mAs. Standard B filter (one of the image reconstruction kernels) was used because it was calibrated by the manufacturer to provide accurate CT Hounsfield numbers and is suitable for routine abdomen, pelvis, and CT angiography scans. It is a recommended filter for clinical routine use. The detailed scanning parameters for this study were summarized in the Table 1.

**Table 1:** The detailed CT scanning parameters utilized in this study

*Adjustable Scanning Parameters*	*Values*
kVp	120 and 140
Collimator setting (mm)	64×0.625
Rotation time (s)	0.5
Pitch	0.06
Reconstruction Filter	Standard B
Reconstruction slice thickness (mm)	3 mm
mAs/slice (effective mAs)	50, 100, 150, 200, 250, 500, 1000, 1500, 2000, 2500, and 3333 (for 120 kVp) or 3000 (for 140 kVp)
Corresponding ${\text{CTDI}}_{\text{vol}}$ (mGy) for 120 kVp scans	3.3, 6.6, 9.8, 13.1, 16.7, 32.7, 65.4, 98.1, 130.8, 168.0, and 216.9
Corresponding ${\text{CTDI}}_{\text{vol}}$ (mGy) for 140 kVp scans	4.9, 9.7, 14.5, 19.3, 24.7, 48.5, 97.0, 145.4, 193.9, 241.6, and 289.2
Reconstruction Algorithms	FBP and ${\text{iDose}}^{4}$ with noise reduction levels 1, 3, & 6
Phantom Diameters (cm)	38, 43, 48, 53, and 58

### C. CT Acquisitions of clinical patients

In addition to the CT scans of the phantom, CT image data from 14 head and neck cancer patients, 10 prostate cancer patients, and 10 gynecology (GYN) cancer patients were collected based on an approved IRB protocol to evaluate the visibility of anatomical structures. The ${\text{iDose}}^{4}$reconstruction algorithm was retrospectively applied to the collected patient cases for further evaluation. Each scan originally reconstructed with FBP was reconstructed using ${\text{iDose}}^{4}$ algorithm with noise reduction levels 1–6. The ${\text{iDose}}^{4}$‐based reconstructed images were compared to the images reconstructed with the traditional FBP technique by two radiation oncologists and using the five‐point scores described in Materials & Methods section F below.

### D. ${\text{iDose}}^{4}$ iterative reconstruction algorithm

The ${\text{iDose}}^{4}$ algorithm, which improves image quality while reducing radiation dose, is achieved through an iterative process as briefly described below. An adaptive linear filter is used on noisy projections in the projection domain followed by quantum mottle noise reduction in the image domain. The dual‐domain processing steps are helpful for suppressing streak artifacts and noise, as well as visualizing underlying anatomical information. The noise filtering in the projection domain by the ${\text{iDose}}^{4}$ algorithm begins with identification of noisy point measurements in each projection. The noise model utilizes photon statistics to identify these data points, which are given a weight penalty through an iterative edge‐preserving diffusion process. In the image domain, the quantum mottle noise distribution within the image volume is estimated and then subtracted using a best‐fit structural model, chosen by the user, as a template. Multifrequency noise subtraction is implemented to maintain noise power spectrum constancy. The suppression effect of the quantum mottle noise in an image can be controlled by the ${\text{iDose}}^{4}$ reconstruction noise reduction levels (1–6), corresponding to a varied reduction range from low to high. By means of the dual‐domain processing, the ${\text{iDose}}^{4}$ reconstruction algorithm can correct bias artifacts and maintain noise power spectrum constancy, while preserving spatial resolution. More details regarding the ${\text{iDose}}^{4}$ reconstruction algorithm can be found in the technical paper provided by Philips and in the literature.^3^, ^9^, ^10^, ^11^

### E. Metrics of image quality and radiotherapy task performance

This comparative study was conducted to quantitatively compare the ${\text{iDose}}^{4}$ iterative reconstruction algorithm to the traditional FBP algorithm based on two image quality‐based metrics, noise and contrast‐to‐noise ratio (CNR), and two radiotherapy task‐based metrics, target CT Hounsfield number and target contouring accuracy (TCA). Considering that the prostate is a critical organ in radiotherapy, the prostate of the anthropomorphic phantom was selected as the target of analysis here. Three adjacent cross‐sectional slices of the prostate were selected, and the same slices were chosen throughout all the reconstructed image sets for fair comparison. The contours of the prostate on the three slices were manually outlined by a trained medical physicist and reviewed by an experienced dosimetrist; radiation oncologists reviewed some selected datasets, as well. The average CT Hounsfield number and noise σ (CT Hounsfield number standard deviation) for each contoured prostate region were measured. $\sigma_{\text{target}}$, which describes the fluctuations in the target signal that may affect target delineation, was defined as

(1) $$\sigma_{\text{target}}=\sqrt{\frac{1}{\left(F-1\right)}\sum_{f=1\Lambda f\in \text{target}}^{F}{\left(CT{\#}_{f}-\bar{CT{\#}_{\text{target}}}\right)}^{2}}$$

where $CT{\#}_{f}$ represents the CT number of each voxel f, ${\bar{CT\#}}_{\text{target}}$ is the average of CT numbers of all the voxels within the target, and *F* is the total voxel numbers within the target. Additionally, the average CT Hounsfield number and noise at the surrounding region outside the prostate were measured in the same way, and was used as the background values. The contrast‐to‐noise ratio (CNR), which describes the ability of the target to be detected against its background, was defined as

(2) $$CNR=\frac{\left|{\bar{CT\#}}_{\text{target}}-{\bar{CT\#}}_{background}\right|}{\sigma_{background}}$$

where ${\bar{CT\#}}_{background}$ is the average of CT Hounsfield numbers of a narrow band of three voxels' width surrounding and outside the prostate, and $\sigma_{background}$ as the background noise, can be calculated using Eq. (1). The CT number, which is used to determine tissue electron density and therefore affects the dose calculation results, was simply defined as the average of CT numbers of the target, and its variations can be evaluated from its changes along with various scan protocols. The TCA of each reconstructed image set was defined as

(3) $$TCA=(C^{\text{target}}\cap C_{ref}^{\text{target}})/\left|C^{\text{target}}\cup C_{ref}^{\text{target}}\right|$$

where $C^{\text{target}}$ is the set of target voxels on the image to be evaluated, and $C_{ref}^{\text{target}}$ is the set of target voxels on reference image. This TCA was defined to consider the target coverage accuracy since it is the most important parameters to evaluate the accuracy of radiation treatment. Confirmed with both radiation oncologists who performed image quality evaluation in this study, the CT image of the 38 cm original phantom scanned with 120 kVp and the greatest available ${\text{CTDI}}_{\text{vol}}$ of 216.9 mGy and reconstructed with FBP algorithm was defined as the reference image. The reference ground truth contour was defined as the manual prostate contour $C_{\text{Ref}}$ delineated on this reference image. A value of one for TCA indicates a complete overlap between the contouring result and the reference contour, while a zero value represents no overlap between two contours. The relationships between the radiation dose, image quality metrics, and task‐based indices were analyzed from the CT images acquired in Materials & Methods section C above.

### F. Qualitative five‐point scoring criteria

We determined a five‐point scoring criteria based on physicians' suggestions for qualitative evaluation of clinical patient images reconstructed by FBP and ${\text{iDose}}^{4}$, respectively. For each case, the FBP‐based and ${\text{iDose}}^{4}$‐based images were reviewed side‐by‐side. The radiation oncologists were allowed to browse through each image series, zoom in and out, and adjust the window settings. They ranked the overall image quality based on the visual conspicuity of critical structures using a five‐point score: 0=totally obscured, no structures identifiable; 1=marked artifacts, questionable recognition; 2=faint anatomic recognition; 3=anatomical recognition with low confidence; 4=anatomical recognition with medium confidence; and 5=anatomical recognition with high confidence for segmentation. All the patients' images were evaluated by two radiation oncologists using the five‐point scores. The preferred dataset and the related ${\text{iDose}}^{4}$ noise‐reduction level (if ${\text{iDose}}^{4}$‐based image was preferred) were also indicated. The final ranking was obtained by averaging the ranks from two radiation oncologists. A Wilcoxon signed‐rank test was performed to analyze the quality ranking and to determine significant differences between the FBP and ${\text{iDose}}^{4}$ reconstructed images. A p‐value of less than 0.05 indicated a statistically significant difference.

## III. RESULTS

### A. Phantom CT simulation data

#### A.1 Noise analysis

**Figure 2:**
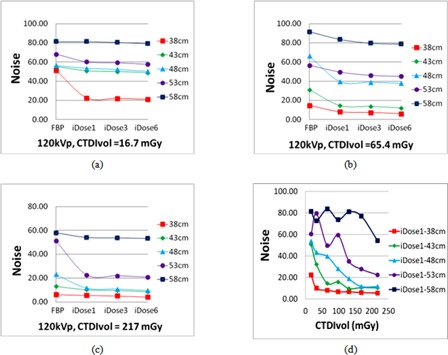
Noise comparison for the prostate; (a)‐(c) image noise plots with respect to different reconstruction algorithms under various radiation doses; (d) image noise plot obtained by the ${\text{iDose}}^{4}$ with noise reduction level 1 algorithm while altering both the phantom thickness and radiation dose, using a 120 kVp setting.

Figure 2 illustrates the relationship of image noise to the reconstruction algorithms and scanning protocols of varying radiation dose levels. Here each reported noise value is the average of measurements on three continuous slices, and the variations are within five HU for all the measurements. For a given phantom size and a given scanning protocol, the ${\text{iDose}}^{4}$ reconstruction algorithm always produced images with lower noise levels than the FBP algorithm, reducing image noise up to 66.1% when compared with the FBP algorithm without considering the clinical acceptance of the images and the changes on special resolutions of images reconstructed by ${\text{iDose}}^{4}$ and FBP reconstruction algorithms, respectively. This highest noise reduction happened on the images that were reconstructed with ${\text{iDose}}^{4}$ reconstruction with noise reduction level 6 on the projection data scanned with the protocol of ${\text{CTDI}}_{\text{vol}}$ of 16.7 mGy on 38 cm sized phantom. As the radiation dose increases, the ${\text{iDose}}^{4}$ algorithm reduces noise more effectively for the larger phantoms. As shown in Figs. 2(a) to 2(c), for the largest phantom size employed in this study (58 cm), the ${\text{iDose}}^{4}$ algorithm was unable to reduce the noise, when compared with FBP, with a 120 kVp scan parameter. This phenomenon occurs even for the highest ${\text{CTDI}}_{\text{vol}}$ (217 mGy) used in this study, as the noise within the prostate was only reduced by 6.6%.

#### A.2 Contrast‐to‐noise ratio (CNR) analysis

**Figure 3:**
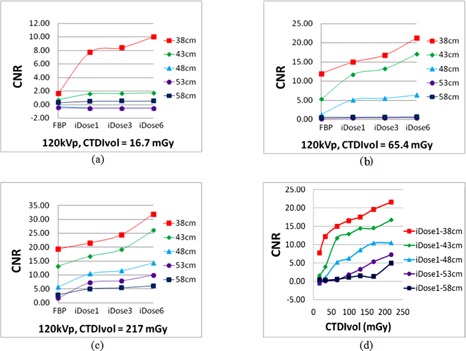
CNR comparison ((a) to (c)) of various reconstruction algorithms; (d) CNRs of a single reconstruction under changes of the phantom thickness and the radiation dose.

Figure 3 illustrates the comparison of the CNR with different scanning doses and image reconstruction algorithms for different phantom thicknesses. The ${\text{iDose}}^{4}$ reconstruction algorithm produced images with a higher CNR than the FBP algorithm. In general, the largest improvement in CNR occurred at noise reduction level 6 for a given phantom size and scan dose. The maximal improvement of the ${\text{iDose}}^{4}$ algorithm over the FBP algorithm occurred for the 38 cm sized phantom. As shown in Fig. 3(a), the CNR is 1.65 when using the FBP algorithm, but increases by 369.5% to 7.74 when using ${\text{iDose}}^{4}$ with noise reduction level 1. For a particular phantom size and scan parameters, the CNR increases with an increasing ${\text{iDose}}^{4}$ noise reduction level. For the largest phantom size used in this study (58 cm), limited CNR improvement was observed when comparing the ${\text{iDose}}^{4}$ algorithm with FBP, for 120 kVp scan parameters. A similar trend was observed from our experiments of the 140 kVp scans, which were not illustrated here.

#### A.3 Target CT number variation analysis

**Figure 4:**
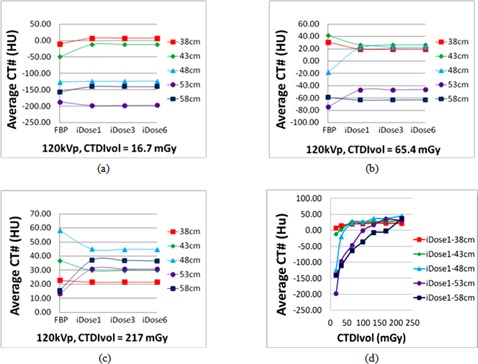
Average CT Hounsfield number comparison; (a)‐(c) plots of the CT number with respect to different imaging parameters for the FBP, the ${\text{iDose}}^{4}$ reconstruction with noise reduction levels 1, 3, and 6; (d) CT number variations obtained by using the ${\text{iDose}}^{4}$ reconstruction with noise reduction level 1 and altering the phantom thickness and the radiation dose.

Accurate and consistent CT Hounsfield numbers are required in order to properly determine the tissue electron density in dose calculations, an important task in radiotherapy. A comparison for the average CT Hounsfield number of the target obtained by different imaging protocols was performed, as illustrated in Fig. 4. Given the same phantom size and radiation dose, the CT number obtained by using the FBP algorithm was different from those acquired by the ${\text{iDose}}^{4}$algorithm. While looking into the ${\text{iDose}}^{4}$ algorithm, for a particular ${\text{CTDI}}_{\text{vol}}$ value and phantom size, a nearly constant CT number was maintained, indicating that ${\text{iDose}}^{4}$ noise reduction level does not affect the consistency of the CT number. Taking the example of the 58 cm sized phantom, the maximum difference of the average CT numbers between the ${\text{iDose}}^{4}$ reconstructions with noise reduction levels 1, 3, and 6 was only a few of Hounsfield units. However, the CT number can vary given different phantom sizes. For a particular reconstruction method, the variations of the CT numbers caused by the phantom size decreased as the ${\text{CTDI}}_{\text{vol}}$ increased. This phenomenon can be observed based on Fig. 4(d), wherein the CT numbers obtained by applying the ${\text{iDose}}^{4}$ reconstruction with noise reduction level 1 for various sizes of the phantom converge progressively as the ${\text{CTDI}}_{\text{vol}}$ increased.

#### A.4 Target contouring accuracy (TCA) analysis

**Figure 5:**
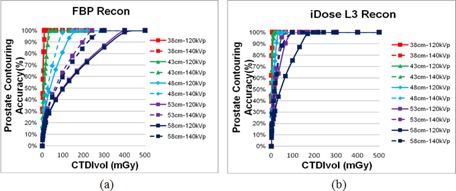
Relationship between the manual prostate contouring accuracy and the ${\text{CTDI}}_{\text{vol}}$ based on the FBP (a) and ${\text{iDose}}^{4}$reconstruction with noise reduction level 3 (b) under various phantom sizes and kVp settings.

Other than CT number variations, the target contouring accuracy has been considered as another important task performance measure since it directly influences the accuracy of the dose‐volume relationship of a particular structure. Figure 5 illustrates the relationship of the TCA for manual contouring with respect to the radiation dose based on the FBP and ${\text{iDose}}^{4}$ reconstruction with noise reduction level 3 for the prostate of the phantom. The TCA for both the FBP and ${\text{iDose}}^{4}$algorithms depended greatly on both the phantom size and the radiation dose. When using the 120 kVp scan protocol, 100% TCA could not be achieved for the large‐size phantoms of 53 and 58 cm diameter, even when a very high ${\text{CTDI}}_{\text{vol}}$ was used in this study (217 mGy). Generally, using the ${\text{iDose}}^{4}$ algorithm can achieve 100% TCA with a lower ${\text{CTDI}}_{\text{vol}}$ than the FBP algorithm requires. It was additionally observed from this analysis that higher radiation doses (i.e., ${\text{CTDI}}_{\text{vol}}$) were needed for larger sized patients in order to achieve the same contouring accuracy as that performed on smaller sized patients. More specifically, for the smallest phantom diameter (38 cm), the prostate could be delineated with 100% accuracy for the lowest‐dose scan (i.e., a ${\text{CTDI}}_{\text{vol}}$ of 16.7 mGy) for both the FBP and ${\text{iDose}}^{4}$ reconstruction with noise reduction level 3.

### B. Patient simulation CT data

The image data for 34 patients, reconstructed by the ${\text{iDose}}^{4}$ algorithm with noise reduction levels 1 to 6, were qualitatively evaluated by two radiation oncologists using the five‐point scoring criteria. Table 2 lists the evaluation results. For all cases, the oncologists preferred to use the simulation CT images reconstructed by the ${\text{iDose}}^{4}$ with moderate noise reduction levels 3–5 for treatment planning. Overall, the ${\text{iDose}}^{4}$ algorithm provided superior radiation dose control while maintaining or improving task performance when compared to FBP. The reader study on patient cases shows that physicians prefer ${\text{iDose}}^{4}$ reconstructed images compared to those from FBP algorithm with overall quality score: 1.21 vs. 3.15, p=0.0022. The qualitative evaluation also indicated that the radiation oncologists chose an optimal and moderate ${\text{iDose}}^{4}$noise reduction level (3 or 4) instead of the highest nose reduction level 6.

**Figure 6:**
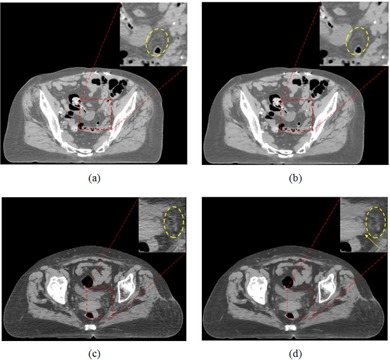
Assessment of anatomy conspicuity and realisticity for the CT images produced by the ${\text{iDose}}^{4}$ algorithm with (a) and (c) noise‐reduction‐level 1, and (b) and (d) noise reduction level 6.

As shown in Fig. 6, although the ${\text{iDose}}^{4}$ algorithm with a higher noise reduction level can improve the CNR, the performed iterative diffusion process (as described in Materials & Methods section D above) achieves edge‐preserving smoothness only from a numerical point of view and lacks consideration of anatomical knowledge and geometrical priors, resulting in unrealistic degradation of spatial resolution (as indicated by the circles) and sharper but jagged edges (as indicated by the arrow), as described in image processing lieterature.^16^, ^17^

**Table 2:** Qualitative evaluation results on clinical patient cases

*Treatment Site*	*Number of the Evaluated Patient Cases*	*Which Reconstruction Algorithm Is Preferred by Physicians? (FBP or* ${\text{IDose}}^{4}$)	*If* ${\text{iDose}}^{4}$ *Algorithm Was Preferred, Which Noise Reduction Level Was Preferred?*
Head & Neck	14	${\text{iDose}}^{4}$	4 to 5
Prostate patients	10	${\text{iDose}}^{4}$	4 to 5
GYN patients	10	${\text{iDose}}^{4}$	3 or 4

**Figure 7:**
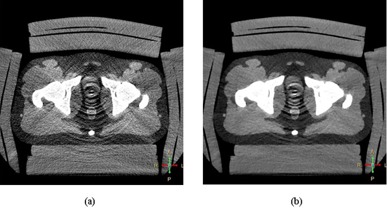
The photon starvation artifacts are shown in images of 53 cm sized phantom scanned with same low‐dose protocol of ${\text{CTDI}}_{\text{vol}}=29.93\;\text{mGy}$, and reconstructed with different reconstruction algorithms: (a) the image is reconstructed with FBP; (b) the image is reconstructed with ${\text{iDose}}^{4}$ and noise reduction level 6.

Figure 7 shows an example of photon starvation artifacts on images of large‐sized phantom scanned with low‐dose protocol. The diameter size of the phantom is 53 cm. The phantom was scanned with low‐dose protocol (${\text{CTDI}}_{\text{vol}}=29.93\;\text{mGy}$). The images were reconstructed with FBP and ${\text{iDose}}^{4}$ algorithms, respectively. It is clear that the photon starvation artifacts exist on both images.

## IV. DISCUSSION

This comparative study has illustrated that the overall clinical efficacy of the FBP and the ${\text{iDose}}^{4}$reconstruction algorithms are dependent on both the patient size and the CT scan parameters. For smaller sized patients, comparably accurate structure contours can be obtained from the images reconstructed by both the FBP and the ${\text{iDose}}^{4}$ algorithms. However, the image noise was lower and the contrast‐to‐noise ratio was improved in the images obtained by using ${\text{iDose}}^{4}$ when compared to the FBP algorithm (see Fig. 2). The noise reduction comparison shown in Fig. 2 is performed to compare only the noise level changes between ${\text{iDose}}^{4}$ and FBP reconstruction algorithms without considering the changes of spatial resolution for these two reconstruction algorithms. In this study, the scanning protocols were varied only on effective mAs and reconstruction algorithms while keeping other scanning parameters, such as slice thickness and reconstruction filter, the same. We have used these protocols to scan the line‐pair phantom provided by the manufacturer and measured the MTF of each images. Interestingly, all the images yielded the same full width at half maximum (FWHM) of line profile as 2.6 lp/cm, and no spatial (high contrast) resolution differences were observed between FBP and ${\text{iDose}}^{4}$reconstructed images in this study. One of our on‐going works is to provide complete investigation on the relation among all scanning parameters to noise level, and spatial and contrast resolution of varied reconstruction algorithms in order to provide guidance on the improvement of contouring accuracy of CT simulations in radiation therapy.

The results shown in Fig. 3 illustrate that, with the ability to reduce image noise while retaining boundary information, the ${\text{iDose}}^{4}$ reconstruction algorithm produces images with a superior CNR when compared with images generated using the FBP method. The results strongly suggested that the CNR was insufficient in distinguishing the performance of CT reconstruction algorithms for large‐size patients. In these cases, task‐based quality assessment, which was stated in the last paragraph of this section, would be required. Our experimental results also indicated that the use of ${\text{iDose}}^{4}$ had no obvious improvement of the clinical task performance (i.e., accuracy of contouring a structure) over FBP for patients with near or below 38 cm diameter (see Fig. 5) even with the lowest‐dose level scanning protocol in this study (${\text{CTDI}}_{\text{vol}}=3.3\;\text{mGy}$). Yet, as the patient size increased, the image quality becomes more dependent upon the selected reconstruction algorithm, which directly influences image characteristics: noise and CNR. In these cases, the ${\text{iDose}}^{4}$ reconstruction can provide more clear appearance of anatomical structures through noise reduction and CNR improvement. Overall, the ${\text{iDose}}^{4}$algorithm provided more accurate structure delineation, compared to the FBP method, for the majority of scan protocols and patient sizes in this study.

In addition, the results shown in Fig. 5 illustrate that an important clinical suggestion can be made based on the proposed task performance‐based metrics rather than the standard image quality‐based metrics such as CNR. For small sized patients it may not be necessary to increase the ${\text{CTDI}}_{\text{vol}}$ in order to achieve improved TCA, even though the CNR may improve, because the TCA is sufficient for a radiotherapy treatment; in that case a radiation dose increase is very likely to cause unnecessary radiation exposure. However, a higher ${\text{CTDI}}_{\text{vol}}$ may be needed for larger size patients in order to achieve 100% TCA.

As shown in Fig. 6, the ${\text{iDose}}^{4}$ reconstruction with noise reduction level 6 may misrepresent delicate signs of pathology and target tissues, from the perception of the oncologists. According to our investigation, this was the major reason the ${\text{iDose}}^{4}$ with noise reduction level 6 was not recommended for use in the clinic. This qualitative study reinforces the assertion that standard image quality characteristics, including the noise and contrast, should not be used as the sole criteria for assessing image quality when considering radiotherapy tasks.

While further considering the effect of CT scan parameters on image quality, the photon starvation artifacts that are caused by a deficit of X‐ray photons in certain areas cannot always be eliminated by increasing the radiation dose, especially for larger sized patients. As shown in Fig. 2(d), for a 38 cm diameter phantom, the image noise can be effectively reduced by increasing the radiation dose from 16.7 mGy to 34 mGy. However, it is impractical to do so for a 58 cm size phantom (and unsafe for patients) because of the effects of photon starvation artifact. In particular, given a low ${\text{CTDI}}_{\text{vol}}$ (e.g., 16.7, 32.7, and 65.4 mGy in this study), the increase of the structure contouring confidence achieved by the ${\text{iDose}}^{4}$ algorithm was very limited for larger sized patient data (i.e., 48 cm diameter or greater in this study). In addition, as shown in Fig. 2(d), the noise level from the 53 cm and 58 cm phantom sizes is not monotonically decreasing. The reason is also due to the irregular photon starvation artifact streaks existing in the images. Low‐dose scanning protocols that inherently cause severe photon starvation artifacts should be avoided in CT simulations, especially of larger sized patients. These results also demonstrated that both reconstruction algorithms cannot physically resolve the photon starvation artifacts, and therefore the radiation dose needs to be increased in the scanning protocol of larger sized patients in order to achieve desirable image quality for contouring purpose.

In order to further analyze the change in CT number and its affect on dose calculation, we performed the quantitative comparison to ascertain whether the treatment plans generated using different radiation dose scans and reconstruction algorithms were clinically equivalent.^11^, ^12^ For the 53 cm sized phantom, five pairs of different dose images (tube potential: 120 kVp; ${\text{CTDI}}_{\text{vol}}$: 32.7, 65.4, 98.1, 130.8, and 168.0 mGy) were reconstructed through the FBP and the ${\text{iDose}}^{4}$ L3 algorithms, respectively. The prostate, bladder, and rectum contours in the reference image (in Material & Methods section E) were firstly mapped to each image dataset. The clinical IMRT treatment plans for prostate cancer were optimized based on a consistent prescription. It was originally determined based on the reference image and reference contours, and recomputed on these five image pairs. The γ dose distribution comparison tool^18^, ^19^ was employed to compute the associated dose distributions. Dose areas with a γ value greater than 0.95 with a 3%/3 mm DTA criterion were considered clinically equivalent. After comparing the reference and test treatment plans, all plans achieved a passing rate of >99.9%. Thus, while different radiation doses and reconstruction algorithms produced images with varying CT number, this variation did not significantly alter the calculated dose distribution.

The dosimetry study explained above indicates that the dose distribution differences were mostly within 1% (at most 3%) of the prescription dose, and were therefore considered as dosimetric equivalent for these five CT image datasets acquired with different dose‐level protocols. This finding can be explained by the Compton interaction of megavoltage therapeutic beams with human body tissues, and was described in one of our previous studies.^(20)^As well‐known in radiation treatment planning, the attenuation of a megavoltage beam in water is less than 3% per cm penetration, which is much less than that of a kilovoltage beam. ^(21)^ In this study, for large sized phantom of 53 cm and 58 cm, the CT number differences on images acquired with the scanning protocols of moderate dose (${\text{CTDI}}_{\text{vol}}$ of 65.4 mGy) and the higher dose protocol (${\text{CTDI}}_{\text{vol}}$ of 216.9 mGy) was less than 100 HU, which translates to a 1 mm uncertainty per cm depth. Without loss of generality, we can assume that the effective prostate region has a size of less than 5 cm, the uncertainly in depth determination is therefore less than 0.5 cm, and it results in a dose calculation uncertainty of 3%/cm×0.5 cm=1.5% at most.^(20)^ As such, the dose distributions are considered as dosimetric‐equivalent no matter which CT image dataset was used for dose computation as long as the same contour was used. As shown in Fig. 4, there was an improvement in CT Hounsfield number consistency when using ${\text{iDose}}^{4}$ reconstruction algorithm with moderate radiation dose‐based scanning protocol. However, as shown in Fig. 7, ${\text{iDose}}^{4}$ reconstruction cannot reduce photon starvation artifacts; very‐low‐dose scanning protocols should be avoided for both FBP and ${\text{iDose}}^{4}$ reconstructions to eliminate or reduce photon starvation artifacts and improve the contouring accuracy. The moderate dose level scanning protocol should be used and adjusted based on patient sizes for CT simulations in radiation therapy.

This study has shown that the ${\text{iDose}}^{4}$ reconstruction algorithm, as compared with the FBP reconstruction algorithm, has the potential to produce CT images which improve radiotherapy task performance. It can reduce noise while maintaining boundary information, maintain CT Hounsfield number consistency, and increase anatomical structure conspicuity. However, the accuracy of structure contouring could decrease when the ${\text{iDose}}^{4}$ noise reduction level increased from 3 to 6, which may lead to a distortion in the image texture of the target. This strongly suggested that improvement of standard image quality characteristics (e.g., the noise and CNR in this study) does not necessarily bring clinical benefits for radiotherapy tasks. As such, either the noise or CNR should not be used as sole criteria for guiding the usage of ${\text{iDose}}^{4}$ reconstruction for CT simulations; instead, the scanning protocol and certain level of anatomical conspicuity (or realisticity) that fulfills radiotherapy task requirements must be jointly considered.^(22)^ On the other hand, for the patient cases analyzed in this study, the radiation dose was chosen by means of the automatic tube current modulation. This comparative study suggested that, even if the scanning dose is reduced to a certain level (depending upon the patient size), as long as the ${\text{iDose}}^{4}$ algorithm is employed to keep the image quality, the radiotherapy tasks could still be accomplished. Our future research will focus on the investigation of task‐based image quality assessment frameworks so as to provide an improved quantitative image selection platform for radiotherapy applications.

## V. CONCLUSIONS

This study evaluated a commercially available Philips ${\text{iDose}}^{4}$ iterative reconstruction technique in radiotherapy environment and compared it to the traditional filter back‐projection (FBP) reconstruction. The ${\text{iDose}}^{4}$ algorithm demonstrated a superior ability over the FBP algorithm in reducing image noise, improving the CNR, maintaining the CT number consistency, and improving target delineation accuracy for both energy levels. We suggest that the ${\text{iDose}}^{4}$algorithm can be used safely and effectively for CT simulation and the subsequent treatment planning. However, the usage of ${\text{iDose}}^{4}$ reconstruction algorithm should be determined by the radiotherapy tasks (e.g., target contour conspicuity and CT number consistency for accurate treatment planning) accompanied with patient‐specific factors (e.g., body size), instead of the common image quality metrics only, such as noise, contrast, and/or CNR. Particularly, our experiments suggested that a moderate noise reduction level of ${\text{iDose}}^{4}$ reconstruction was preferred for the radiotherapy tasks, as an overly high noise reduction level is most likely to cause unrealistically smooth CT images with mosaic artifacts.

## ACKNOWLEDGMENTS

We would like to especially thank Dr. Lifeng Yu from the Mayo Clinic for his helpful discussion and insight regarding task‐based image quality assessment.

## COPYRIGHT

This work is licensed under a Creative Commons Attribution 3.0 Unported License.
